# Assaying proline hydroxylation in recombinant collagen variants by liquid chromatography-mass spectrometry

**DOI:** 10.1186/1472-6750-12-51

**Published:** 2012-08-17

**Authors:** S W Polly Chan, John Greaves, Nancy A Da Silva, Szu-Wen Wang

**Affiliations:** 1 Department of Chemical Engineering and Materials Science, University of California, Irvine, CA, 92697, USA; 2 Department of Chemistry, University of California, Irvine, CA, 92697, USA

**Keywords:** Hydroxyproline, Liquid chromatography-mass spectrometry, LC-MS assay, Recombinant collagen

## Abstract

**Background:**

The fabrication of recombinant collagen and its prescribed variants has enormous potential in tissue regeneration, cell-matrix interaction investigations, and fundamental biochemical and biophysical studies of the extracellular matrix. Recombinant expression requires proline hydroxylation, a post-translational modification which is critical for imparting stability and structure. However, these modifications are not native to typical bacterial or yeast expression systems. Furthermore, detection of low levels of 4-hydroxyproline is challenging with respect to selectivity and sensitivity.

**Results:**

We have developed a new liquid chromatography-mass spectrometry (LC-MS) method to evaluate proline hydroxylation in recombinant collagen. This assay was tested in different *Saccharomyces cerevisiae* expression systems to evaluate the effect of gene ratio between prolyl-4-hydroxylase and collagen on the extent of hydroxylation. These systems used a human collagen III gene that was synthesized *de novo* from oligonucleotides. The LC-MS assay does not require derivatization, uses only picomoles of sample, and can measure proline hydroxylation levels in recombinant and native collagen ranging from approximately 0% to 40%. The hydroxylation values obtained by LC-MS are as accurate and as precise as those obtained with the conventional method of amino acid analysis.

**Conclusions:**

A facile, derivatization-free LC-MS method was developed that accurately determines the percentage of proline hydroxylation in different yeast expression systems. Using this assay, we determined that systems with a higher collagen-to-hydroxylase gene copy ratio yielded a lower percentage of hydroxylation, suggesting that a specifically balanced gene ratio is required to obtain higher hydroxylation levels.

## Background

Collagen is the most abundant class of proteins in the extracellular matrix and it demonstrates dynamic interactions with its biological microenvironment [1]. Therefore, fabrication of *de novo*, synthetic collagen in which specific sequences can be prescribed has tremendous potential in tissue engineering, drug delivery applications, and fundamental biological studies of the extracellular matrix. Towards this end, two challenges exist: (1) the synthesis of genes encoding the collagen-mimetic polymers which contain repeating amino acid sequences, and (2) the hydroxylation of prolines in a recombinant system. To address the first constraint, our research group has developed a platform that yields genes encoding for full-length, modular collagen and its variants. This approach allows mixing-and-matching of specific functional amino acid sequences from the different families of collagens and enables introduction of non-native sequences at defined locations, combinations, and number of occurrences [2].

We are addressing the second challenge, recombinant expression coupled with post-translational proline hydroxylation, by designing and optimizing genetic modifications in *Saccharomyces cerevisiae*. Proline hydroxylation is particularly important, as it imparts stability and structure to collagen. However, bacterial and yeast expression systems do not natively perform this post-translational modification, and must be engineered to produce prolyl-4-hydroxylase (α and β subunits). These systems, however, tend to yield biopolymers with lower levels of proline hydroxylation, ranging from 0.5% to 38% proline hydroxylation in recombinant collagens produced in *S. cerevisiae*[2-4] and 44.2 − 47.2% in the best *P. pastoris* reported systems [5,6]. In comparison, fibrillar human collagens from native tissues show 42–54% hydroxylation [7,8].

Given the large possible range of values, we needed an accessible and facile assay that can determine the level of proline hydroxylation in future libraries of recombinant collagen and its variants. Such an assay should also use relatively small amounts (pmol) of sample, require minimal processing and derivatization, and potentially enable high-throughput scale-up. As others have noted, however, detection of 4-hydroxyproline (HYP) is particularly challenging with respect to both selectivity and sensitivity [9].

To address these difficulties, analytical methods for HYP often require derivatization [10-13]. In fact, the conventional method of determining the percentage of proline hydroxylation, amino acid analysis (AAA), measures the concentration of amino acid residues after derivatization with a fluorescent probe, such as ninhydrin [14,15]. However, to assay relatively small quantities (picomole), a sensitive and expensive fluorescence detector is required on the liquid chromatography system. Protocols using radioisotopes have also been developed [16], but the logistics of using radioactive compounds are inconvenient if appropriate research infrastructure is not in place.

Our aim was to develop a rapid method to quantify HYP without further derivatization by utilizing mass spectrometry (MS) instrumentation that would be accessible in most research institutions. Mass spectrometry protocols requiring no additional chemical reaction have been reported using hydrophilic interaction chromatography (HILIC) [13] and tandem (LC-MS/MS) mass-spectrometry with multiple reaction monitoring (MRM) [9,17]. Our method to quantify the amounts of proline (PRO) and HYP in different collagen samples uses a simple and standard reversed-phase liquid chromatograph coupled to a single analyzer time-of-flight MS (LC-MS) and requires no sample derivatization.

We applied this LC-MS assay to engineered *S. cerevisiae* systems that we expected would yield various levels of proline hydroxylation. These yeast strains contained different collagen to prolyl-4-hydroxylase gene ratios on plasmid vectors. To determine the reliability of this LC-MS assay, these hydroxylation results were compared to conventional AAA.

## Methods

### Quantification of proline and hydroxyproline

#### Liquid Chromatography – Mass Spectrometry (LC-MS) method

The LC-MS consisted of an Agilent 1100 instrument and a Waters LCT Classic mass spectrometer in an open access user facility. The liquid chromatography separations used a solvent system of 2% acetonitrile and 0.2% acetic acid in water (solvent A) and 0.2% acetic acid in acetonitrile (solvent B), with a 45-minute solvent program that reached 25% B at 25 min followed by a rapid ramp to 95% B to remove unwanted compounds from the column. Ten-μl samples dissolved in acetonitrile/water (50:50 v/v) were injected onto a Phenonenex Luna 5 μ C18 100 Å 150 mm long × 2.0 mm internal diameter column connected directly to the mass spectrometer. Electrospray ionization (ESI) was used in positive ion mode.

#### Determination of standard curves

Calibration standards were D-proline (Aldrich) and trans-4-hydroxy-L-proline (Aldrich), and the internal standard (IS) was glycyl phenylalanine (Sigma). D-proline could be used in place of L-proline because the two stereoisomers give identical elution times and calibration curves. To obtain calibration curves, we injected different concentrations of PRO and HYP which were dissolved in acetonitrile/water (50:50 v/v) containing 0.5 μg/ml glycyl phenylalanine. The PRO, HYP, and IS peaks were identified based on their masses and retention times. Reconstructed ion chromatograms (RIC) for the protonated PRO and HYP and major fragments were plotted. The internal standard RIC included the molecular species, its fragments, and the acetonitrile adduct ions. Calibration curves were obtained by plotting the area ratios of the PRO/IS and HYP/IS against the PRO and HYP concentrations. Three sets of calibration curves were determined immediately before and/or after each set of collagen samples was run on the LC-MS, and an average was taken of the linear regression equations. Linearity was acceptable (R^2^ greater than 0.990) for PRO concentrations between 0.2 μg/ml and 5 μg/ml and for HYP concentrations between 0.2 μg/ml and 1.5 μg/ml. A secondary confirmation was obtained by diluting analytical samples by a factor of two and ensuring that the resulting concentrations were similarly halved.

### Vector and strain constructions

Haploid *Saccharomyces cerevisiae* BY4741-ΔTRP1 (strain BY4741 with a TRP1 deletion: *MATa his3Δ1 leu2Δ0 met15Δ0 ura3Δ0 Δtrp1:: KanMX*) [18] was used to express the modular collagen and the human prolyl-4-hydroxylase. *E. coli* strain XL1-Blue (Stratagene) was used for plasmid maintenance and amplification. Human prolyl-4-hydroxylase subunit genes p4Ha and p4Hb were cloned into a low copy CEN/ARS vector and co-transformed with the 2 μ modular collagen plasmid into BY4741-ΔTRP1 as described previously [19]. The p4Ha gene was cloned under the *GAL10* promoter and the p4Hb gene with a yeast invertase (SUC2) leader sequence was cloned under the *GAL1* promoter on the same CEN/ARS plasmid. The modular collagen gene was under the control of the *S. cerevisiae GAL1* promoter [2]. To test the effect of balancing the collagen-to-hydroxylase gene copy ratio, the collagen gene was placed on a CEN/ARS plasmid. The GAL1p-MCOL gene cassette was excised using HindIII from YEpMCOL [2], inserted into YCplac22 [20] to create YCpMCOL-trp, a low-copy CEN/ARS vector. This vector was co-transformed with the CEN/ARS hydroxylase vector into BY4741-ΔTRP1. As a negative control strain (no hydroxylase present), YEpMCOL was transformed into BY4741-ΔTRP1 alone without the hydroxylase plasmid.

### Collagen expression and purification

Strains carrying the dual plasmids were inoculated into selective medium and cultured as described in Chan et al. [2]. Cells were lysed and their background protein was digested with pepsin. Recombinant collagen was purified from the digested protein by cationic exchange chromatography as described in Chan et al. [2].

### Preparation and analysis of FPLC-purified collagen samples

Samples of dried bovine collagen (5 to 12 μg; Millipore) and FPLC-purified recombinant collagen samples (40 to 80 μg) were hydrolyzed in 6 N hydrochloric acid (Pierce) vapor at 150°C for 90 minutes under vacuum [15]. The hydrolysate was resuspended with acetonitrile/water (50:50 v/v) containing 0.5 μg/ml of the IS and injected onto the LC-MS system. For comparison, AAA was performed by the Biopolymer Lab at UCLA (http://www.mbi.ucla.edu/services/biopolymer-laboratory). For AAA, protein samples were hydrolyzed in 6 N (vapor-phase) HCl for 22 hours at 110°C. The free amino acids were then derivatized with 6-aminoquinoly-N-hydroxysuccinimidyl carbamate (AQC, Waters), a fluorescent amino-reactive probe, and the products were fractionated and quantified by reversed-phase HPLC.

### Preparation and analysis of SDS-PAGE-purified collagen samples

To investigate the feasibility of isolating collagen from SDS-PAGE for analysis, SDS-PAGE loading buffer (6×) was added to collagen samples (totalling 320 μl), heated at 95°C for 5 minutes, and run on a 7% SDS-PAGE gel. The gel was washed with water, stained in 0.1% Coomassie Blue R-250 (Fisher) dissolved in 10% acetic acid/50% methanol/40% water, and then destained in 10% acetic acid/50% methanol/40% water to determine band locations. The collagen bands were excised, placed in a glass vial, further destained by washing three times in 10% acetic acid/50% methanol/40% water, and stored at 4°C overnight. To extract the collagen, acetonitrile (Fisher, Optima LC-MS grade, 100 μl) was added to the vial that was then sonicated in a water bath for 20 minutes at 40 kHz. The acetonitrile was recovered and a second extraction with 50 μl fresh acetonitrile was performed. The combined acetonitrile extracts were dried under nitrogen in a LC-MS vial and then subjected to hydrolysis as described for FPLC-purified samples. The products were resuspended with 0.1 ml acetonitrile:water (50:50 v/v) containing 0.5 μg/ml IS and analyzed by LC-MS.

### Percentage hydroxylation calculation

**Figure 1:**
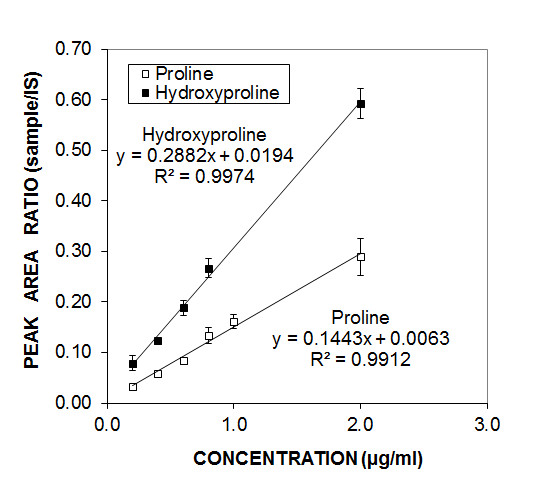
Proline and hydroxyproline standard curves.

The HYP present in collagen is the product of *in vivo* hydroxylation of PRO residues [21], and we defined the percent hydroxylation of proline to be (moles of HYP)/(moles of HYP + moles of PRO) × 100%. The percent hydroxylation was calculated using concentrations obtained from the LC-MS data. Final values presented in Table 1 are the result of averaging triplicate measurements from 2–3 independent experiments for LC-MS, and measurements of triplicate experiments for AAA. The reported percent hydroxylation values obtained by AAA have the background chemical noise (0.18%) subtracted; this was obtained from the yeast strain containing the collagen gene, but without hydroxylase genes (negative control). The background signal observed in the LC-MS for this negative control was less than 1% relative to readings of any of the HYP standards (Figure 1) and was therefore considered insignificant. 

**Table 1:** Percent hydroxylation of proline in bovine collagen III and in recombinant human collagen III

	**LC-MS Assay (%)**	**Amino Acid Analysis (%)**
Bovine collagen III (as-received)	38 ± 1.1	37 ± 2.7
Bovine collagen III (extracted from SDS-PAGE)	36 ± 3.4	n/a
Recombinant human collagen III	N.D.*	N.D.*
No hydroxylase (p4Ha, p4Hb) genes		
Collagen on 2-micron plasmid		
Recombinant human collagen III	5.8 ± 0.5	5.2 ± 1.3
CEN/ARS–p4Ha–p4Hb with SUC2 signal		
Collagen on 2-micron plasmid		
Recombinant human collagen III	12 ± 1.8	12^§^
CEN/ARS–p4Ha–p4Hb with SUC2 signal		
Collagen on CEN/ARS plasmid		

* "N.D." denotes no significant signal of hydroxyproline above background noise.

^§^Only 1 AAA result available.

## Results and Discussions

### LC-MS method quantifies proline and hydroxyproline

The peaks for proline (PRO), hydroxyproline (HYP), and the internal standard (IS) were identified from injecting pure samples in the LC-MS system. The choice of the internal standard was based on stability, similarity of chemical nature, molecular mass (when compared with PRO and HYP), and commercial availability. Elution times were 1.6 minutes for HYP, 1.65 minutes for PRO, and 2.25 minutes for the IS, glycyl phenylalanine. These elution times were expected for non-derivatized amino acids on the reversed-phase column. The peak area ratios between the fixed amount of internal standard and the two amino acids, proline and hydroxyproline, yielded calibration curves that increased linearly with the amino acid concentrations (Figure 1). Typical linear regression equations were y = 0.144x + 0.006 for PRO and y = 0.288x + 0.019 for HYP, with the coefficients of regression (R^2^) greater than 0.994. Therefore, for unknown protein hydrolysates, the peak area ratios for the two amino acids against the internal standard can be used to determine the amino acid relative concentrations.

### LC-MS assay gives values comparable to conventional amino acid analysis over full range of proline hydroxylation for collagen

**Figure 2:**
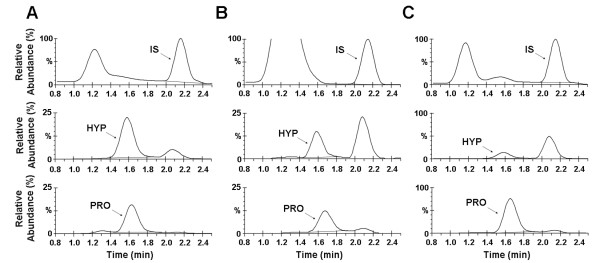
**Reconstructed ion chromatograms of the hydrolyzed collagen samples.** Samples were separated by reversed-phase liquid chromatography and detected by ESI mass spectrometry. **(A)** Bovine collagen III, as received, **(B)** Bovine collagen III extracted from SDS-PAGE, and **(C)** Recombinant human collagen III. Rows: Top, IS = internal standard; Middle, HYP = hydroxyproline; Bottom, PRO = proline.

To evaluate our LC-MS method, we examined percent hydroxylation of commercial bovine collagen III and recombinant collagen from two *S. cerevisiae* strains engineered to produce prolyl-4-hydroxylase. From prior amino acid analysis (AAA), we expected that the percent hydroxylation for the recombinant systems would yield intermediate values that are less than those of native (bovine) collagen and therefore enable us to probe the full range of possible hydroxylation values. Figure 2 shows reconstructed ion chromatographs (RIC) of representative collagen samples. Based on the mass and elution times, the PRO, HYP, and IS peaks were identified. The integrated areas under the PRO and HYP peaks were divided by the integrated area of the IS and their relative concentrations were obtained using the respective standard curves. Percentage proline hydroxylation was then determined as described in Methods.

The percentage proline hydroxylation values are summarized in Table 1. For native bovine collagen III (positive control), the percent hydroxylation of proline was determined by our LC-MS assay to be 38 ± 1.1% (n = 3 separate samples, with each sample injected in triplicate). The percentage hydroxylation of bovine collagen was also determined by amino acid analysis (AAA), which showed 37 ± 2.7% hydroxylation. These values are consistent with a previously-reported value of 41% hydroxylation for bovine collagen as determined by AAA [22]. For the negative control, we placed a collagen gene on a 2-micron plasmid in yeast that did not contain the prolyl hydroxylase genes. As expected, neither the LC-MS nor AAA methods yielded hydroxylation signals beyond background chemical noise.

To examine the ability of the LC-MS assay to determine intermediate hydroxylation levels, we evaluated collagen produced from a 2-micron vector (high copy number) and a CEN/ARS (low copy number) vector. In both cases, the hydroxylase enzymes were co-expressed with collagen and carried on a CEN/ARS vector. For these recombinant samples, our LC-MS method obtained percent hydroxylation of 5.8 ± 0.5% for collagen on the 2-micron (high copy number) vector and 12 ± 1.8% for the CEN/ARS (low copy number) plasmid. In comparison, AAA of these samples gave 5.2 ± 1.3% and 12% hydroxylation, respectively. Collectively, our data shows that for determining the percentage of hydroxylated proline, the LC-MS method yields values equivalent to the conventional strategy of AAA.

Furthermore, the precision for the hydroxylation measurements using our LC-MS method for bovine, recombinant (2-micron), and recombinant (CEN/ARS) collagen samples were 2.9%, 7.9%, and 15% relative standard deviation (RSD), respectively. In contrast, using AAA, the RSD for bovine collagen and recombinant collagen (2-micron) were 7.3% and 23%, respectively. This demonstrates that the LC-MS method is also at least as precise, if not more so, as conventional AAA.

Many AAA systems have a sensitivity of 1 pmole [15], with a recommended minimum of 50 pmoles for quantitative analysis. Our experience with AAA has shown that this minimum level of 50 pmoles is especially critical for the quantification of HYP. Below this level, the elution time of HYP in AAA is very close to that of the hydrolysis by-product, 6-aminoquinoline, resulting in overlapping baselines in the chromatogram. In our LC-MS assay, the lower limit of quantification for the pure amino acids is approximately 15 pmole on column for both PRO and HYP. In addition, because reconstructed ion chromatographs are used, there is no problem of an overlap with 6-aminoquinoline. Therefore, the amount of samples needed for both our LC-MS assay and AAA are comparable in magnitude, with the LC-MS assay requiring slightly less sample.

### Evaluation of SDS-PAGE sample purification method

The recombinant human collagen variants presented in Table 1 were purified by fast protein liquid chromatography (FPLC), as described in Methods. However, FPLC can be time-consuming and not practical for larger-scale screens of protein variants, so we investigated whether collagen that was extracted from SDS-PAGE could yield reasonable protein hydroxylation values using the LC-MS assay. Success in quantifying HYP from these extracts would enable analysis of samples without development of lengthy purification protocols.

Although purified by the commercial source, the bovine collagen still shows impurity bands when run on SDS-PAGE gels. The percent hydroxylation for bovine collagen III extracted from SDS-PAGE gave 36 ± 3.4%, which was close to the values measured from the as-received samples (38 ± 1.1%). However, the RSD (an indicator of repeatability) of bovine collagen extracted from SDS-PAGE was 9.4%, which is higher than the 2.9% RSD of as-received collagen, suggesting that the additional steps and reagents required in SDS-PAGE extraction may decrease the precision of our method.

For recombinant human collagen extracted from SDS-PAGE gels, we found that LC-MS could indeed detect the significantly lower levels of hydroxylation present (relative to fully hydroxylated bovine collagen); however, these results were not as accurate or precise as for FPLC-purified samples. This demonstrates that extraction from SDS-PAGE could potentially be used to screen protein candidates to identify desired levels of hydroxylation, but chromatography-based purification may be necessary for in-depth analysis.

### Balanced enzyme to collagen gene ratio is required for higher proline hydroxylation levels

We have assayed the proline hydroxylation levels of recombinant collagen produced in two dual-plasmid yeast strains. The first strain overexpressed the collagen using a 2 μ plasmid and produced a 5.8% hydroxylated protein. The second strain expressed collagen from a stable, low copy CEN/ARS plasmid, and in this case the hydroxylation increased to 12%. In both cases, the two hydroxylase genes were carried on a second CEN/ARS plasmid. Therefore, the two strains have different collagen to hydroxylase gene ratios, and this is reflected in the percent hydroxylation of the collagen produced. When the copy number of the collagen gene was high relative to the hydroxylase genes, we obtained a lower level of hydroxylation.

Native collagen has a significantly greater proline hydroxylation level (38% for bovine collagen III) than our systems reported here. Based on the findings from the two dual plasmid systems, the percentage hydroxylation should increase if the hydroxylase and collagen expression levels are appropriately balanced. We expect that further increasing the number of p4H genes or their expression levels should increase hydroxylation. In light of this, our current work includes integrating the two hydroxylase subunits and the collagen gene cassettes into the yeast genome to control tightly the collagen-to-prolyl hydroxylase gene copy ratio. Our facile LC-MS assay will be very helpful in determining the percentage hydroxylation of these integrated strains and identifying the construct with hydroxylation levels closest to native collagen.

## Conclusions

We have developed an LC-MS method for determining the percent hydroxylation of collagen from native and recombinant sources. In our studies, this method has been used to analyze native collagen and recombinant collagen (expressed in yeast) with hydroxylation percentages ranging from 0 to approximately 40%. Our LC-MS method is as accurate and precise as the conventional amino acid analysis, and has been used to assess hydroxylation levels for different recombinant *S. cerevisiae* strains producing collagen. The results demonstrate the importance of balancing collagen and hydroxylase expression levels in the yeast host. Overall, this new LC-MS method is a rapid and reliable analytical method for screening proteins with varying levels of proline hydroxylation, and it provides an alternative method to conventional amino acid analysis.

## Competing interests

The authors declare that they have no competing interests.

## Authors' contributions

SWPC performed molecular cloning, design of plasmids, expression and purification of proteins, and design and running LC-MS assays. JG participated in the design of the LC-MS method. NAD and SWW participated in the design and coordination of the overall study. All authors contributed to the writing of the manuscript, and all authors have read and approved the final manuscript.

## References

[B1] Gelse K Poschl E Aigner T Collagens - structure, function, and biosynthesis Adv Drug Deliv Rev 2003 55 12 1531 1546 10.1016/j.addr.2003.08.002 14623400

[B2] Chan SWP Hung SP Raman SK Hatfield GW Lathrop RH Da Silva NA Wang SW Recombinant human collagen and biomimetic variants using a de novo gene optimized for modular assembly Biomacromolecules 2010 11 6 1460 1469 10.1021/bm100052y 20481478

[B3] Vaughan PR Galanis M Richards KM Tebb TA Ramshaw JA Werkmeister JA Production of recombinant hydroxylated human type III collagen fragment in Saccharomyces cerevisiae DNA Cell Biol 1998 17 6 511 518 10.1089/dna.1998.17.511 9655244

[B4] Toman PD Chisholm G McMullin H Gieren LM Olsen DR Kovach RJ Leigh SD Fong BE Chang R Daniels GA Production of recombinant human type I procollagen trimers using a four-gene expression system in the yeast Saccharomyces cerevisiae J Biol Chem 2000 275 30 23303 23309 10.1074/jbc.M002284200 10801837

[B5] Vuorela A Myllyharju J Nissi R Pihlajaniemi T Kivirikko KI Assembly of human prolyl 4-hydroxylase and type III collagen in the yeast Pichia pastoris: formation of a stable enzyme tetramer requires coexpression with collagen and assembly of a stable collagen requires coexpression with prolyl 4-hydroxylase EMBO J 1997 16 22 6702 6712 10.1093/emboj/16.22.6702 9362485 PMC1170275

[B6] Nokelainen M Tu HM Vuorela A Notbohm H Kivirikko KI Myllyharju J High-level production of human type I collagen in the yeast Pichia pastoris Yeast 2001 18 9 797 806 10.1002/yea.730 11427962

[B7] Uitto J Collagen polymorphism – isolation and partial characterization of alpha-1 (I)-trimer molecules in normal human skin Arch Biochem Biophys 1979 192 2 371 379 10.1016/0003-9861(79)90105-X 434832

[B8] Chung E Miller EJ Collagen polymorphism - characterization of molecules with chain composition [Alpha-1(III)]3 in human tissues Science 1974 183 4130 1200 1201 10.1126/science.183.4130.1200 4812351

[B9] Kindt E Gueneva-Boucheva K Rekhter MD Humphries J Hallak H Determination of hydroxyproline in plasma and tissue using electrospray mass spectrometry J Pharm Biomed Anal 2003 33 5 1081 1092 10.1016/S0731-7085(03)00359-5 14656599

[B10] Prockop DJ Udenfriend S A specific method for the analysis of hydroxyproline in tissues and urine Anal Biochem 1960 1 3 228 239 10.1016/0003-2697(60)90050-6 13738134

[B11] Husek P Rapid derivatization and gas-chromatographic determination of amino-acids J Chromatogr 1991 552 1–2 289 299

[B12] Marfey P Determination of d-amino acids. 2. Use of a bifunctional reagent, 1,5-difluoro-2,4-dinitrobenzene Carlsberg Res Commun 1984 49 6 591 596 10.1007/BF02908688

[B13] Langrock T Czihal P Hoffmann R Amino acid analysis by hydrophilic interaction chromatography coupled on-line to electrospray ionization mass spectrometry Amino Acids 2006 30 3 291 297 10.1007/s00726-005-0300-z 16622599

[B14] Eastoe JE Amino acid composition of mammalian collagen and gelatin Biochem J 1955 61 4 589 600 13276342 10.1042/bj0610589 PMC1215839

[B15] Ozols J Deutscher MP Amino acid analysis Methods in Enzymology 1990 Academic Press, New York 587 601

[B16] Juva K Prockop DJ Modified procedure for assay of H3-or C14-Labeled hydroxyproline Anal Biochem 1966 15 1 77 83 10.1016/0003-2697(66)90249-1 5959433

[B17] Colgrave ML Allingham PG Jones A Hydroxyproline quantification for the estimation of collagen in tissue using multiple reaction monitoring mass spectrometry J Chromatogr A 2008 1212 1–2 150 153 18950772 10.1016/j.chroma.2008.10.011

[B18] Winzeler EA Shoemaker DD Astromoff A Liang H Anderson K Andre B Bangham R Benito R Boeke JD Bussey H Functional characterization of the S-cerevisiae genome by gene deletion and parallel analysis Science 1999 285 5429 901 906 10.1126/science.285.5429.901 10436161

[B19] Chan SWP Fabrication of modular human collagen and collagen variants in yeast. PhD thesis 2012 Department of Chemical Engineering, University of California, Irvine

[B20] Gietz RD Sugino A New yeast-Escherichia-coli shuttle vectors constructed with in vitro mutagenized yeast genes lacking 6-base pair restriction sites Gene 1988 74 2 527 534 10.1016/0378-1119(88)90185-0 3073106

[B21] Berg RA Determination of 3-hydroxyproline and 4-hydroxyproline Methods Enzymol 1982 82 372 398 7078444 10.1016/0076-6879(82)82074-0

[B22] Steven FS Jackson DS Purification and amino acid composition of monomeric and polymeric collagens Biochem J 1967 104 2 534 539 6048794 10.1042/bj1040534 PMC1270616

